# Percutaneous Sclerotherapy of Postoperative Lymphoceles: A Systematic Review and Meta-analysis of Different Sclerosant Agents

**DOI:** 10.1007/s00270-026-04371-0

**Published:** 2026-02-17

**Authors:** Marcello Lippi, Alessandro Posa, Gianmarco Quarta, Alessandro Maresca, Vincenzo Vingiani, Pierluigi Barbieri, Anna Rita Scrofani, Andrea Contegiacomo, Alessandro Cina, Roberto Iezzi

**Affiliations:** 1 Department of Diagnostic Imaging, Radiation Oncology, and Hematology, A. Gemelli University Hospital Foundation, IRCCS, Largo A. Gemelli 8, 00168 Rome, Italy; 2 Università Cattolica del Sacro Cuore, Sede di Roma, Largo F. Vito 1, 00168 Rome, Italy; 3 Department of Radiology, Hospital of Bolzano (SABES-ASDAA), Teaching Hospital of Paracelsus Medical University (PMU), 5 Böhler Street, 39100 Bolzano, Italy

**Keywords:** Lymphocele, Postoperative lymphocele, Sclerotherapy, Sclerosant agents, Percutaneous drainage, Systematic review, Meta-analysis

## Abstract

**Purpose:**

This study aims to provide a meta-analysis of relevant outcomes related to various sclerosant agents used in percutaneous lymphocele sclerotherapy.

**Materials and Methods:**

A systematic review, registered on PROSPERO (Record CRD420251180687), was performed using PubMed, Scopus, and Web of Science, including studies reporting any kind of postoperative lymphoceles treated with a sclerosant agent. Searches were updated to November 2025. Pooled success, recurrence, and complication rates were estimated for each sclerosant agent, and differences across agents were explored. The risk of bias was assessed using Methodological Index for Non-Randomized Studies, while the certainty of the evidence was assessed using the Grading of Recommendations, Assessment, Development and Evaluation.

**Results:**

Sixteen studies were included—fifteen retrospective and one prospective—reporting on different sclerosant agents: ethanol, povidone-iodine, OK-432, fibrin glue, polidocanol, and doxycycline. A total of 335 lymphoceles were studied. Ethanol showed the most consistent results, with high clinical success (97.4%, 95% CI 82.6–99.7%) and low recurrence rates across the studies (6.7%, 95% CI 3.1–14.2%). The other sclerosant agents yielded similar results but with a lower grade of evidence certainty. Minor complication rates were low (12.2%), consisting predominantly of mild and self-limiting adverse events; only two major complications were reported. Limitations were heterogeneity among studies and predominance of retrospective designs.

**Conclusions:**

Percutaneous sclerotherapy yielded favorable results with all agents; however, ethanol is the best-characterized agent, with consistent outcomes and moderate certainty of evidence. High-quality, prospective research is needed in order to choose the best sclerosant agent and standardize the procedure.

**Level of Evidence:**

Level IV, systematic review of retrospective non-comparative studies.

**Graphical Abstract:**

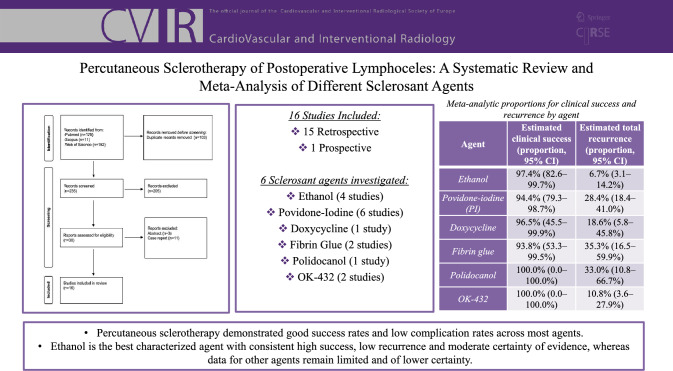

## Introduction

Lymphoceles are common postoperative complications after major surgery, resulting from disruption of lymphatic channels and subsequent accumulation of lymphatic fluid [1]. They are frequently asymptomatic and are found incidentally on postoperative imaging [2, 3]. However, depending on their size and location, they can cause significant clinical issues, such as ureteral obstruction, limb swelling, impaired wound healing, and worsening of renal graft function [4].

Lymphoceles are often detected through imaging, combined with biochemical analysis data to differentiate them from other postoperative collections [2–7].

Small or asymptomatic lymphoceles may resolve spontaneously or remain stable without intervention, whereas symptomatic or enlarging collections typically require treatment [1, 8]. Although surgical marsupialization was historically effective, it is associated with higher postoperative morbidity [1]. Percutaneous drainage is now widely accepted as first-line therapy, but recurrence remains common [1]. To reduce recurrence rates, sclerotherapy has been introduced as an adjunctive treatment, involving instillation of a sclerosant after drainage to induce inflammation and fibrosis of the cavity [7, 9].

Clinical outcomes after sclerotherapy may be influenced by lymphocele-related factors such as size, clinical presentation, and treatment burden. Despite the widespread use of percutaneous sclerotherapy, the choice of sclerosant agent remains largely empirical. This systematic review and meta-analysis therefore aims to consolidate the existing evidence on the clinical efficacy, recurrence, and adverse events associated with the principal sclerosant agents used in the percutaneous treatment of postoperative lymphoceles, by estimating pooled outcome rates for each agent and exploring differences across agents.

## Materials and Methods

### Protocol and Registration

This systematic review was registered on PROSPERO (Record CRD420251180687).

### Search Strategy and Data Extraction

**Fig. 1:**
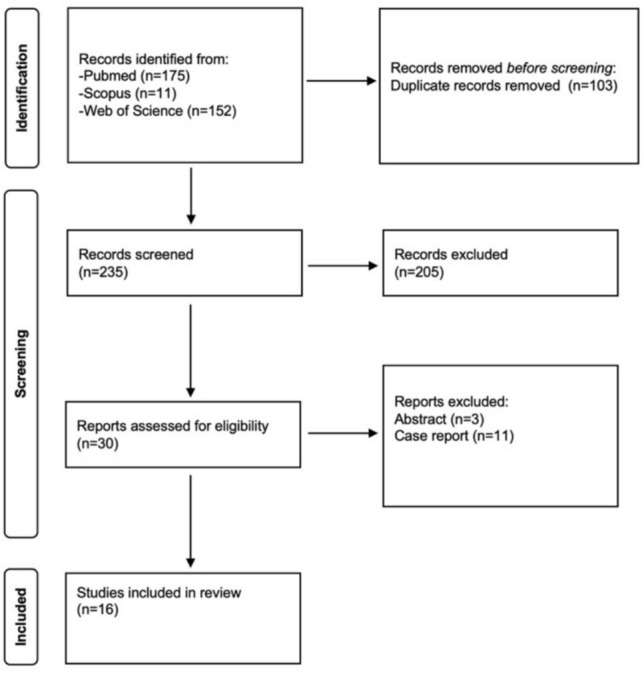
PRISMA flowchart

A comprehensive search was performed across PubMed, Scopus, and Web of Science using the terms: (lymphocele OR lymphatic leak OR lymphatic leakage) AND (sclerotherapy OR sclerosant OR ethanol OR alcohol OR povidone iodine OR iodopovidone OR doxycycline OR OK-432 OR picibanil) AND (percutaneous OR drainage OR catheter). No filters regarding study design or publication type were applied initially. The search was limited to studies involving humans and articles published in English. Two reviewers (ML and GQ) independently screened all titles and abstracts for eligibility; then a full-text review of the shortlisted articles was performed. Any disagreements were resolved by two additional reviewers (RI and AP). This study used the Preferred Reporting Items for Systematic Reviews and Meta-Analyses (PRISMA) standards (Fig. 1).

### Study Selection and Eligibility

Study inclusion was based on prospective or retrospective studies reporting percutaneous sclerotherapy for postoperative lymphoceles, regardless of the surgical procedure or sclerosant type. Studies were included if the treated postoperative collection was explicitly described by the authors as a lymphocele. Studies primarily addressing other types of lymphatic leakage, such as chylous collections, chyloperitoneum, or persistent lymphorrhea without a defined collection, were excluded. Abstracts, case reports with fewer than five patients and studies lacking outcome data were excluded. Studies involving lymphangiography embolization or surgical methods were also excluded.

### PICO

PICO framework was used for clinical question as follows:

- *Population *Adult patients (≥ 18 years) with postoperative lymphoceles following any major surgery. Studies that included mixed adult–pediatric populations were included when outcomes were reported for the overall cohort, whereas pediatric-only studies were excluded.
- *Intervention *Percutaneous sclerotherapy with any individual sclerosant agent, such as: ethanol, povidone-iodine, doxycycline, OK-432, fibrin glue, others if reported.
- *Comparator *Another sclerosant agent or no direct comparator (when studies describe a single agent).
- *Outcomes *Clinical success, recurrence and complications.

### Data Items

Postoperative lymphocele was defined as a localized postoperative lymphatic fluid collection identified by imaging and, when available, biochemical analysis of aspirated fluid. In older series lacking detailed diagnostic criteria, studies were included when the clinical and procedural context was consistent with a postoperative lymphocele as reported by the original authors. To ensure data uniformity, clinical success was defined as symptom resolution with cessation of lymphatic drainage and radiologic resolution or a stable residual cavity without surgical intervention at ≥ 3 months of follow-up. Recurrence was defined as symptomatic or radiologic reaccumulation requiring repeat percutaneous or surgical treatment and was classified as early (< 3 months) or late (≥ 3 months). Recurrence was not considered clinical failure if resolved with repeat sclerotherapy; failure was defined by the need for surgery. Complications were classified as major or minor according to the need for intervention, hospitalization, or resulting morbidity. Follow-up was calculated from surgery to the last clinical or imaging assessment. Surgical settings were recorded for descriptive purposes only. Study characteristics and outcomes were summarized in tables, and forest plots were generated for the quantitative synthesis.

### Statistical Analysis

We first produced descriptive summaries by sclerosing agent (ethanol, povidone-iodine, doxycycline, fibrin glue, polidocanol, and OK-432), aggregating study-level counts to obtain crude pooled proportions of clinical success, total recurrence, and complications. For clinical success and total recurrence, we then conducted single-arm meta-analyses of proportions using generalized linear mixed models (GLMM). In these models, the number of events and the total number of lymphoceles were modelled as binomial outcomes, and the type of sclerosing agent was included as a categorical moderator. Results are presented as pooled proportions with 95% confidence intervals (CIs) back-transformed from the logit scale. Between-study heterogeneity was quantified using τ^2^ and I^2^, and the significance of between-agent differences was assessed with a Wald-type test of moderators (QM), testing the null hypothesis that all agents have the same pooled effect and the alternative that at least one agent differs significantly. To evaluate the robustness of the QM analysis, a sensitivity analysis was performed excluding sclerosing agents represented by a single study.

Complication rates are reported descriptively by agent using exact binomial 95% CIs rather than formal meta-analytic comparisons, because of the low rate of complications. All analyses were conducted in R (version 2025.05.1 + 513). Subgroup analysis based on surgical setting or lymphocele characteristics was considered; however, these variables were inconsistently reported and could not be reliably linked to agent-specific outcomes, precluding meaningful stratified analyses.

### Quality Assessment

The Methodological Index for Non-Randomized Studies (MINORS) [10] was used to evaluate the methodological quality (Risk of Bias) of the included studies because they were non-comparative and ROBINS-I was not applicable. Quality is considered poor when the score is 8 or less, moderate when the score is between 9 and 14, and good when the score is between 15 and 16. Additionally, the overall certainty of evidence was assessed using the Grading of Recommendations, Assessment, Development and Evaluation (GRADE) approach [11]. The GRADE tool rates the certainty of evidence as high, moderate, low, or very low based on factors such as risk of bias, inconsistency, indirectness, imprecision, and publication bias.

## Results

The literature screening started from a total of 338 studies. A total of 14 studies, retrieved in full-text assessment, were excluded. Three were excluded because they were abstracts and eleven because they were case reports, which did not meet the predefined study design eligibility criteria.

Sixteen non-comparative studies [7, 9, 12–25] (335 lymphoceles) were included in the quantitative synthesis, evaluating ethanol (4 studies, 113 lymphoceles), povidone–iodine (6 studies, 130 lymphoceles), doxycycline (1 study, 21 lymphoceles), fibrin glue (2 studies, 23 lymphoceles), polidocanol (1 study, 12 lymphoceles) and OK-432 (2 studies, 36 lymphoceles) (Table 1). Crude pooled rates by agent are shown in Table 2.

**Table 1:** Included studies

References	Agent	Lymphoceles	Clinical success (≥ 3 mo)	Early recurrence ≤ 3 mo	Late recurrence > 3 mo	Total recurrence	Follow-up (months)	Major complications (n)	Minor complications (n)	Lymphocele size/volume	Sclerotherapy sessions (n)
Zuckerman [21]	Ethanol	32	29 (91%)	N.R	N.R	2 (6%)	25 (mean; 2 days–64 mo)	0	5	Size 2 × 2 × 2 cm to 19 × 14 × 11 cm; initial drained volume 2–1200 mL	Mean 7 sessions; median 4 sessions
Sawhney [7]	Ethanol	13	13 (100%)	1	0	1 (7%)	15.7 (mean; 1–41 mo)	0	0	Initial drained mean volume 220 mL)	N.R
Tasar [22]	Ethanol	18	X	1	0	1 (5%)	N.R	0	5	Mean size 6 × 8 × 10 cm; mean volume 230 mL	N.R
Akhan [20]	Ethanol	50	49 (98%)	4	0	4 (8%)	25.8 (mean, 2–64 mo)	0	6	Mean lymphocele volume 329 mL	Single session 20%; multisessions 80%
Gilliland [16]	PI	9	8 (89%)	0	0	0 (0%)	3 (min)	0	0	Drained volume 50–5000 mL	N.R
Cohan [25]	PI	7	6 (86%)	3	1	4 (57%)	6 (min)	0	1	N.R	N.R
Rivera [19]	PI	19	19 (100%)	6	0	6 (32%)	52 (mean, 7–144 mo)	0	0	Mean size 7 cm (range 5–10 cm)	N.R
Montalvo [18]	PI	17	17 (100%)	3	0	3 (18%)	6 (mean, 5 weeks– 2 y)	0	1	Drained mean volume 250 mL	1 session 82%2 sessions 18%
Alago [9]	PI	18	18 (100%)	2	0	2 (11%)	22.6 (mean)	0	N.R	Mean cavity size 295 cc	N.R
Baboudjian [17]	PI	60	33 (55%)	N.R	N.R	27 (45%)	33 (median, 14–60 mo)	1	7	Median size 10 cm (IQR 6.7–12.0 cm)	Median 3 sessions (IQR 3–3)
Caliendo [23]	Doxycycline	21	20 (95%)	N.R	N.R	4 (19%)	9 (mean, 1–18 mo)	0	5	Mean initial size of 7 × 7x10 cm; mean initial volume 325 mL	1 session 82%; 2 sessions 18%
Chin [12]	Fibrin glue	8	6 (75%)	N.R	N.R	4 (50%)	27.7 (mean, 4–44 mo)	0	3	Size: 5.2 × 3.6 × 2.8 cm to 10 × 10 × 11 cm	1 session 50%; 2 sessions 50%
Silas [13]	Fibrin glue	15	15 (100%)	4	0	4 (27%)	16 (mean, 195–856 days)	0	0	N.R	1 session 67%; 2 sessions 13%; 3 sessions 20%
Klode [24]	Polidocanol	12	12 (100%)	4	0	4 (33%)	10 (mean, 1–55 mo)	0	0	N.R	1 session in 67%; 2 sessions in 25%; 3 sessions 8%
Uyulmaz [14]	OK-432	20	X	1	0	1 (5%)	N.R	0	0	N.R	Mean 2.5 ± 1.2 sessions
Kashiwagi [15]	OK-432	16	16 (100%)	3	0	3 (19%)	17 (mean, 2.5–48.8 mo)	1	8	Mean initial volume 616 mL	1 session 81%; 2 sessions 19%

PI, povidone-iodine; mo,months; Min, minimum; N.R, not reported; X, not applicable

**Table 2:** Crude pooled rates

Agent	Total studies (n)	Studies with FU ≥ 3 mo (n)	Studies with FU < 3 mo (n)	Total N lymphoceles	Clinical success ≥ 3 mo n (%)	Total recurrence (n)	Major complications n (%)	Minor complications n (%)
Ethanol	4	3	1	113	91/95 (96%)	8/113 (7%)	0/113 (0%)	16/113 (14%)
Povidone-iodine	6	6	0	130	101/130 (78%)	42/130 (32%)	1/130 (1.0%)	9/130 (7%)
Doxycycline	1	1	0	21	20/21 (95%)	4/21 (19%)	0/21 (0%)	5/21 (24%)
Fibrin glue	2	2	0	23	21/23 (91%)	8/23 (35%)	0/23 (0%)	3/23 (13%)
Polidocanol	1	1	0	12	12/12 (100%)	4/12 (33%)	0/12 (0%)	0/12 (0%)
OK-432	2	1	1	36	16/16 (100%)	4/36 (11%)	1/36 (3%)	8/36 (22%)

mo, months; FU, follow-up; n, number

Fifteen studies were retrospective [7, 9, 12–24] and only one prospective [25]. Of these, 14 studies reported sufficient follow-up (≥ 3 months) to assess clinical success according to the predefined criteria, while two studies [14, 22] lacked adequate follow-up and were excluded from success-rate analysis.

In every study, the sclerosant agent was instilled through a drainage catheter (range 8–14 Fr).

Follow-up was heterogeneous: not reported in two studies [14, 22], described as a minimum of 3 months by Gilliand et al. and minimum of 6 months by Cohan et al. [16, 25].

The remaining studies report the mean follow-up (range 9–52 months across the studies).

The surgical settings were heterogeneous across studies: lymphoceles occurred after kidney transplantation in 6 studies [12, 13, 17–19, 22], after different types of oncologic surgeries in 7 studies [7, 9, 16, 20, 21, 23, 25] and after lymphadenectomy in 3 studies [14, 15, 24].

Lymphocele size/volume and number of sclerotherapy sessions were variably reported across studies (Table 1), with size described using linear dimensions, volumetric measurements, or drained volumes, and session data reported either categorically or as summary statistics. Due to heterogeneity in definitions and reporting formats, these variables could not be quantitatively incorporated into the analyses.

### Clinical Success

**Fig. 2:**
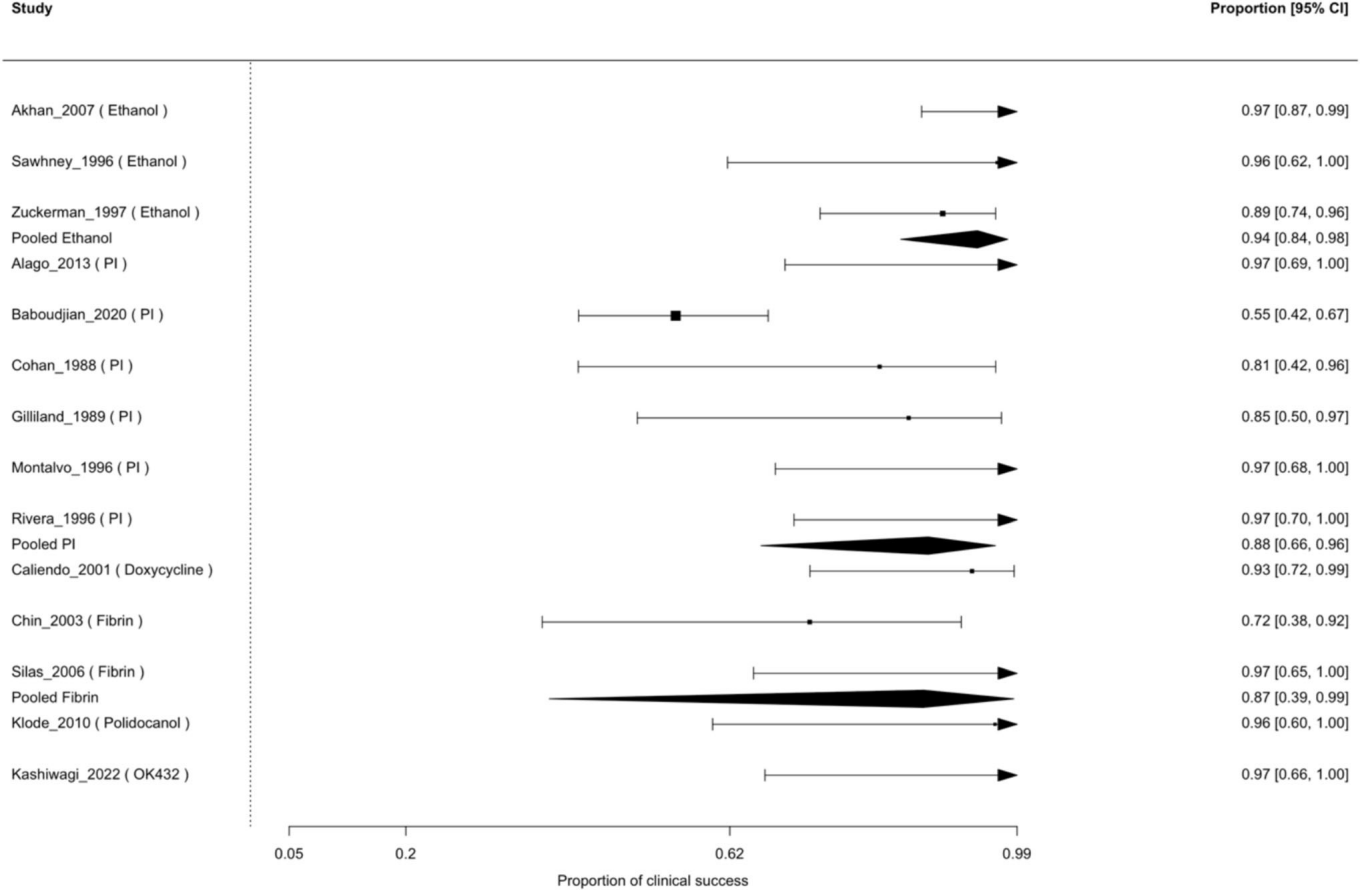
Forest plot of clinical success

In the random-effects GLMM for clinical success, 14 studies with complete success data were included. The pooled probability of clinical success was highest for ethanol (97.4%, 95% CI 82.6–99.7%) and remained high for doxycycline (96.5%, 45.5–99.9%), povidone-iodine (94.4%, 79.3–98.7%) and fibrin glue (93.8%, 53.3–99.5%) (Table 3). For polidocanol and OK-432, the model yielded pooled success estimates of 100%, but with extremely wide confidence intervals (0–100%) because each agent was represented by a single small series with no failures. Figure 2 shows the forest plot of clinical success among sclerosant agents. Residual heterogeneity was moderate (τ^2^ = 2; I^2^ =68%), and the test of moderators indicated significant differences between agents (QM(df = 6) = 33.5, *p* < 0.0001). In the sensitivity analysis restricted to agents with at least two contributing studies, between-agent differences remained statistically significant (QM(df = 4) = 27.41, *p* < 0.0001), with substantial residual heterogeneity (τ^2^ = 2.41; I^2^ = 72%).

**Table 3:** Model-based estimated outcome rates by sclerosant agent

Agent	Estimated clinical success (proportion, 95% CI)	Estimated total recurrence (proportion, 95% CI)
Ethanol	97.4% (82.6–99.7%)	6.7% (3.1–14.2%)
Povidone-iodine (PI)	94.4% (79.3–98.7%)	28.4% (18.4–41.0%)
Doxycycline	96.5% (45.5–99.9%)	18.6% (5.8–45.8%)
Fibrin glue	93.8% (53.3–99.5%)	35.3% (16.5–59.9%)
Polidocanol	100.0% (0.0–100.0%)	33.0% (10.8–66.7%)
OK-432	100.0% (0.0–100.0%)	10.8% (3.6–27.9%)

Estimates for polidocanol and OK-432 are based on very small series with no observed failures; therefore, confidence intervals span from 0 to 100%, reflecting extreme imprecision due to sparse data

### Recurrence

**Fig. 3:**
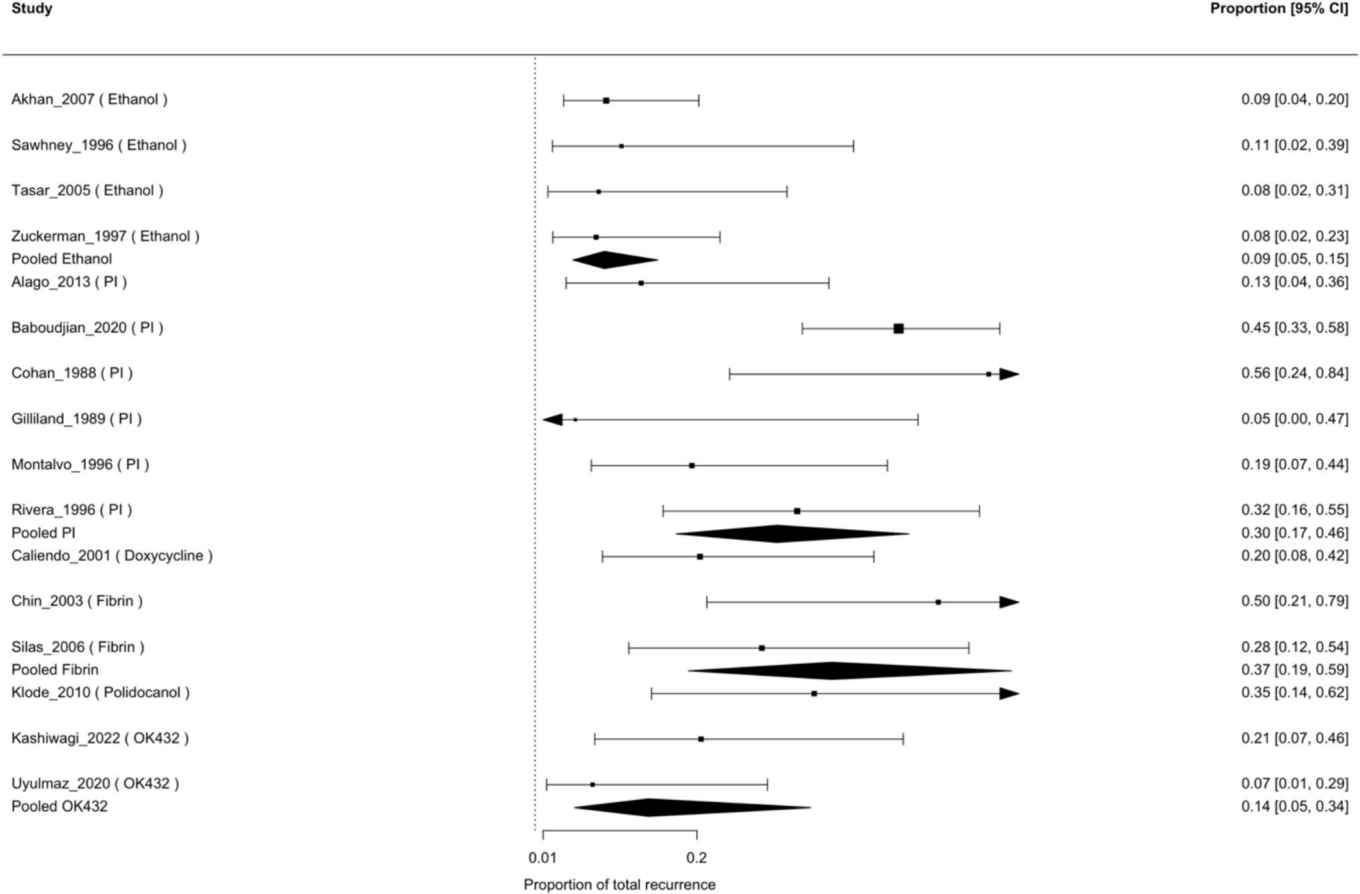
Forest plot of total recurrence

Early recurrence (< 3 months) was the predominant form of recurrence when reported, with only one study documenting one late (> 3 months) recurrence [25]. Three studies analyzed total recurrence, not differentiating late from early recurrence [12, 17, 23]. For total recurrence, all 16 studies contributed to the GLMM. Therefore, separate quantitative meta-analyses for early versus late recurrence were not feasible due to insufficient and inconsistent reporting, so total recurrence was used as the most consistently available outcome. The pooled recurrence probability was lowest for ethanol (6.7%, 95% CI 3.1–14.2%) and OK-432 (10.8%, 3.6–27.9%), intermediate for doxycycline (18.6%, 5.8–45.8%) and povidone-iodine (28.4%, 18.4–41.0%), and highest for fibrin glue (35.3%, 16.5–59.9%) and polidocanol (33.0%, 10.8–66.7%) (Table 3). Figure 3 shows the forest plot of total recurrence among sclerosant agents. Residual heterogeneity was low to moderate (τ^2^ = 0.13; I^2^ = 24%), and the agent effect remained statistically significant (QM(df = 6) = 66.3, *p* < 0.0001). In the sensitivity analysis excluding single-study agents, the test of moderators remained highly significant (QM(df = 4) = 54.61, *p* < 0.0001), with moderate residual heterogeneity (τ^2^ = 0.19; I^2^ = 31%).

### Complications

Major complications were rare: Baboudjian et al. reported one case of acute renal failure due to intraperitoneal diffusion of povidone-iodine, and Kashiwagi et al. described a small bowel fistula. Minor complications, mainly transient fever, mild pain, or local inflammatory reactions, occurred at low rates across all agents (41/335; 12.2%) and did not require further invasive treatment. No study reported treatment-related mortality. Agent-specific minor complication rates ranged from 6.9% for povidone-iodine to approximately 14–24% for ethanol, fibrin glue, doxycycline and OK-432.

### Data Quality

**Fig. 4:**
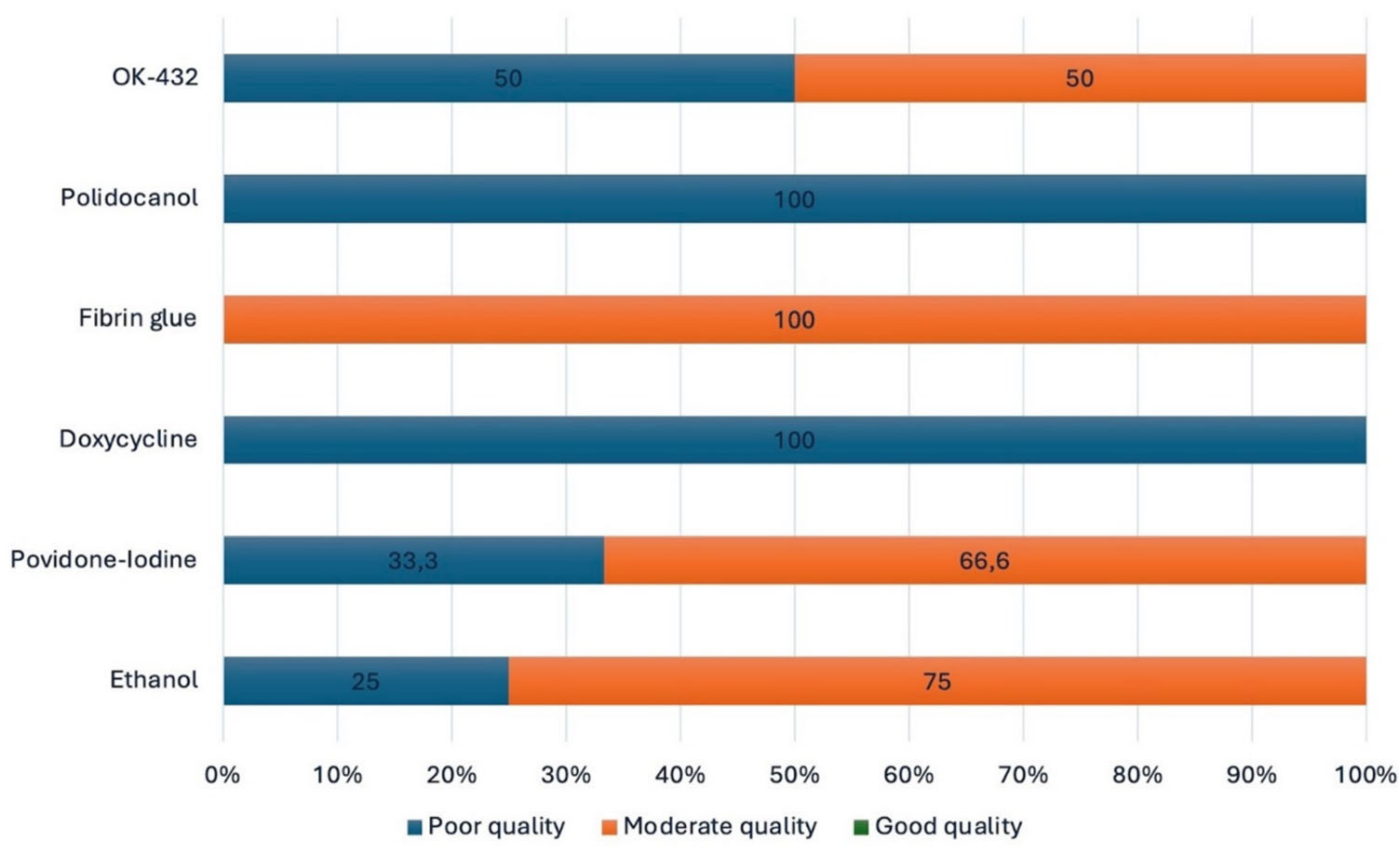
MINORS assessment

MINORS assessment of risk of bias showed mostly low–moderate quality scores (Fig. 4; range 7–13/16). These limitations led to a low GRADE certainty for most agents, except ethanol, which achieved moderate certainty due to consistent high success across larger cohorts.

## Discussion

This systematic review demonstrates that percutaneous sclerotherapy is a highly effective minimally invasive treatment for postoperative lymphoceles, with high overall clinical success rates across different sclerosant agents. These findings are consistent with a previous systematic review by Ten Hove et al. [1], which showed that catheter drainage combined with sclerotherapy achieved significantly higher success rates than drainage alone or repeated aspiration, with outcomes comparable to lymphatic embolization. More recently, Moussa et al. [26] reported higher first-attempt success and shorter drainage times with lymphatic embolization compared with sclerotherapy, while complication rates were similar, suggesting embolization as a potential alternative approach [27].

To date, no clinical guidelines exist for the management of postoperative lymphoceles, largely due to limited high-quality evidence and heterogeneity in clinical practice. In addition, no randomized controlled trials have directly compared surgical and percutaneous approaches.

This study shows that ethanol is the best-characterized sclerosant, demonstrating consistent efficacy and recurrence rates below 10%, though minor complications are not uncommon. Povidone-iodine appears comparably effective, although evidence is less consistent. Doxycycline, evaluated in only a single series, also achieved high efficacy without relevant adverse events, but evidence is too limited for firm conclusions. More recently, OK-432 has emerged as a promising agent, achieving very high technical and clinical success and shorter treatment duration, with the main adverse event being transient fever and one reported major complication (bowel fistula).

Selection of an optimal sclerosant agent should consider not only efficacy and complication rates but also pain and tolerability. However, pain-related outcomes were inconsistently reported and not assessed using standardized definitions or scales, precluding quantitative synthesis. Where described, ethanol and doxycycline were associated with transient procedural discomfort and inflammatory symptoms [20–22], whereas fibrin glue and polidocanol were generally well tolerated with minimal discomfort [12, 13, 24].

Future research should prioritize multicenter, prospective, adequately powered studies with standardized outcome definitions, including intraprocedural pain assessment and long-term follow-up. Direct comparisons between sclerosants, as well as comparative trials involving sclerotherapy, surgery, and embolization, are needed.

This review has several limitations. Most included studies were retrospective, single-center series with heterogeneous surgical settings, treatment protocols, lymphocele characteristics, and follow-up durations. Sample sizes were small, and complications were variably classified. Importantly, pooled recurrence estimates should be interpreted with caution, as recurrence did not necessarily represent definitive clinical failure and follow-up and recurrence timing were inconsistently reported. Consequently, differences in recurrence rates across agents should be considered exploratory rather than confirmatory.

## Conclusions

Percutaneous sclerotherapy is an effective, minimally invasive method for treating postoperative lymphoceles, generally showing high success rates and low complication rates across most agents. However, the procedure is still not standardized. Ethanol is the best-characterized sclerosant agent, with consistently high success rates, low recurrence, and moderate certainty of evidence. In contrast, data for other agents remain limited and of lower certainty.

The lack of standardized reporting of lymphocele characteristics, severity grading, and treatment burden across primary studies limits the level of clinically actionable detail available and highlights an important area for improvement in future research.

## Abbreviations

GLMM: Generalized linear mixed model
CI: Confidence interval
QM: *Q* test for moderators
PRISMA: Preferred reporting items for systematic reviews and meta-analyses
MINORS: Methodological index for non-randomized studies
GRADE: Grading of recommendations, assessment, development and evaluation
